# Leveraging Environmental Contact and Sensor Feedback for Precision in Robotic Manipulation

**DOI:** 10.3390/s24217006

**Published:** 2024-10-31

**Authors:** Jan Šifrer, Tadej Petrič

**Affiliations:** 1 Department of Automatics, Biocybernetics and Robotics, Jožef Stefan Institute, Jamova cesta 39, 1000 Ljubljana, Slovenia; tadej.petric@ijs.si; 2 Jožef Stefan International Postgraduate School, Jamova cesta 39, 1000 Ljubljana, Slovenia

**Keywords:** robotic manipulation, environmental contact, inverse kinematics, quadratic programming, precision control, sensor integration

## Abstract

This paper investigates methods that leverage physical contact between a robot’s structure and its environment to enhance task performance, with a primary emphasis on improving precision. Two main approaches are examined: solving the inverse kinematics problem and employing quadratic programming, which offers computational efficiency by utilizing forward kinematics. Additionally, geometrical methods are explored to simplify robot assembly and reduce the complexity of control calculations. These approaches are implemented on a physical robotic platform and evaluated in real-time applications to assess their effectiveness. Through experimental evaluation, this study aims to understand how environmental contact can be utilized to enhance performance across various conditions, offering valuable insights for practical applications in robotics.

## 1. Introduction

Industrial robots are widely recognized for their exceptional repeatability and precision, essential attributes in many manufacturing and production environments [1]. However, the rise of collaborative robots (cobots) has shifted the focus toward prioritizing safety in human–robot interaction [2,3,4]. Although cobots are engineered for safe, close-quarters collaboration with humans [5], this emphasis on safety can sometimes come at the cost of precision, particularly in tasks requiring intricate and dynamic movements [6,7]. Maintaining high accuracy becomes even more challenging when cobots are required to perform precise dynamic actions, as their inherent mechanical joint stiffness—stemming from their structural design—can limit the robot’s ability to achieve the desired motion accuracy [7].

To address this limitation, we propose a novel solution that leverages physical contact to enhance precision. Drawing inspiration from human behavior [8,9], where individuals often stabilize their hands against a surface during tasks requiring fine motor control [10], we recognize that this technique is commonly used in everyday activities like writing or assembling objects to improve accuracy. Additionally, studies show that children frequently stabilize their forearms when learning new motor skills, highlighting the role of physical stabilization in achieving precision [11]. This principle is equally critical in specialized tasks, such as pipetting in laboratories or performing delicate surgical procedures, where even minor deviations can lead to significant consequences. Our approach applies this concept by positioning the robot to intentionally contact a stable surface during operation, reducing the effects of joint stiffness and mechanical inaccuracies, thereby significantly enhancing precision in dynamic tasks. By integrating this approach, cobots can achieve a balance between safety and precision, expanding their utility in scenarios where both attributes are equally important. The implementation of this strategy could revolutionize how cobots are used in various industries, enhancing their versatility and efficiency in tasks that require both collaborative safety and high precision.

Leaning on a surface introduces a multi-task problem for robotic mechanisms [12], requiring the robot to handle multiple objectives simultaneously. We first explore methods that enable robots to perform such tasks in parallel. For example, one task may involve the robot’s end effector following a designated trajectory, while the second task requires the robot to maintain contact with a surface, applying a predefined force. Before examining the methods that enable this kind of multi-task execution, it is essential to note that all tasks can be described within two distinct operational spaces.

The paper is organized as follows. In Section 2, we provide the background and explore methods for solving inverse kinematics as well as the optimization approach. Section 4 presents the experimental validation of the different methods. Finally, a critical review and conclusions are discussed in Section 5.

## 2. Review of Methods

To establish a foundation for our contributions and elucidate the distinctions in our approach, we begin by revisiting the fundamental concepts of joint space and task space. This review serves to emphasize the critical differences between these spaces and to provide essential context for explaining the methodology and its advancements. By reaffirming these core principles, we aim to demonstrate how the proposed approach not only builds upon but also extends current techniques in robotic control.

The first space, referred to as joint space, is represented as $q=[q_{0},\;q_{1},\;\cdots ,\;q_{N-1}]$, where *N* denotes the number of joints in the mechanism. Joint space uniquely defines the configuration of the robotic mechanism. The task for the robotic mechanism, however, is described in the task space, which provides an alternative representation of the robot’s behavior. Typically, task space is defined in Cartesian coordinates; however, it can be arbitrarily defined in other coordinate systems depending on the task requirements. Task space is represented as $x=[x_{1},\;x_{2},\;\cdots ,\;x_{M}]$, where *M* denotes the number of degrees of freedom (DOF) required for the task. Given that N>M, the task space description has redundancy, allowing for multiple joint configurations to achieve the same task. These two spaces are mathematically related through the forward kinematics Equation [13]

(1) x=p(q).

The forward kinematic equation can be expressed as a non-linear function p. By differentiating this equation, an expression is obtained that describes how changes in the configuration of the robotic mechanism affect the task space. This relationship is captured by the following equation

(2) $$\dot{x}=J\dot{q},$$

where J(q) represents the Jacobian matrix, which relates the velocity in joint space $\dot{q}$ to the velocity in task space $\dot{x}$. The Jacobian provides a linear approximation of how the task space variables change in response to small changes in the joint space configuration. This equation is critical for controlling the mechanism, as it allows for direct manipulation of the task space through joint space adjustments.

For the effective control of the robotic mechanism along a desired trajectory in task space, it is essential to compute the necessary changes in joint configuration that correspond to specific task space movements. This leads to the consideration of the inverse kinematic equation, which forms the foundation for solving the problem of determining joint space adjustments to achieve the desired task space behavior. The inverse kinematics formulation is given as follows:

(3) $$\dot{q}=J^{*}\dot{x}.$$

In this context, $J^{*}$ represents a generalized inverse operator, typically employed to address the non-invertibility of the Jacobian matrix to address redundancy [14]. As a consequence of the redundancy inherent in such robotic systems, there exist numerous methods for controlling the robot. These control strategies can be broadly classified into two categories, as follows:

- Methods for solving inverse kinematics;
- Methods based on optimization approach.

### 2.1. Methods for Solving Inverse Kinematics

The first approach focuses on methods that solve Equation (2). The primary distinction between these methods lies in how they compute an approximation for the inverse of the Jacobian matrix J. Initially, different approaches for calculating the inverse of the Jacobian matrix J will be examined. Following this, methods for controlling the robot mechanism based on these approximations will be explored.

In the context of redundant manipulators, the Moore–Penrose pseudo-inverse is employed for its capability to manage rank-deficient matrices. It is particularly useful when solving Equation (2), which can be interpreted as finding a solution that minimizes the distance $∥J\dot{q}-\dot{x}∥$. If the Jacobian matrix J undergoes Singular Value Decomposition (SVD) as

(4) $$J=U\Sigma V^{T}=\sum_{i=1}^{n}\sigma_{i}u_{i}v_{i}^{T},$$

then the solution to the minimization problem $\min∥J\dot{q}-\dot{x}∥$ can be derived using the pseudo-inverse, and is given by

(5) $$J^{+}\dot{x}=V\Sigma^{+}U^{T}\dot{x}=\sum_{i=1}^{n}v_{i}\frac{1}{\sigma_{i}}u_{i}^{T}\dot{x}.$$

As discussed in [15], Equation (5) reveals that the pseudo-inverse operator introduces discontinuities near singular configurations of the robot. This implies that the Jacobian Pseudo-inverse (JP) algorithm tends to produce large $\dot{q}$ values when the system approaches singularity. A useful metric for determining proximity to a singular value is the condition number, defined as

(6) $$\kappa \left(J\right)=\frac{\sigma_{1}}{\sigma_{n}},\;\mathrm{where}\;\sigma_{1}>\cdots >\sigma_{n}>0,\;\mathrm{and}\;\mathrm{as}\;\sigma_{n}\to 0.$$

The condition number κ(J) approaches infinity as $\sigma_{n}\to 0$, indicating that the Jacobian Pseudo-inverse (JP) algorithm produces large $\dot{q}$ values when the system is under high conditioning [16]. This behavior is particularly problematic near singular configurations, where the smallest singular value $\sigma_{n}$ becomes very small, leading to numerical instability in the control of the manipulator.

In contrast, the Jacobian Transpose (JT) algorithm can avoid the large gains associated with the JP method, as noted in [17], but the underlying conditioning issue persists. Consequently, both the JP and JT approaches are vulnerable to high condition numbers, which can result in numerical instability and excessively large joint velocity magnitudes, particularly as the manipulator nears singular configurations.

Additionally, reducing the global gain is not an effective strategy, as it uniformly decreases the gain in all directions, including those in which the robot should move efficiently. To address the issue of excessive gains, Selective Damping (SD) has been proposed as a solution [18]. This method targets specific directions, thereby mitigating the problem of large joint velocities. However, while SD effectively manages gain-related issues, it does not fully resolve problems related to singularities, such as the loss of rank in the Jacobian matrix and the occurrence of algorithmic singularities in robotic manipulators.

To mitigate the effects of singularities, several alternative algorithms have been proposed. One such method is Jacobian Damping (JD) [19], where a small diagonal term λ is added when computing the pseudo-inverse of the Jacobian matrix. This approach increases all singular values, helping to avoid the singularity problem. However, it also decreases the overall accuracy of the solution due to the uniform increase in singular values.

Another method, the Filtered Jacobian algorithm (JF) [20], introduces a damping term λ that varies depending on the proximity of the robot to a singularity. This approach allows for more adaptive control of the robot near singular configurations. Both the JD and JF algorithms reduce the conditioning of the system, thereby avoiding singularities, but this comes at the cost of significant precision loss, particularly when the robot operates near singular values [16].

The Error Damping (ED) approach [21] involves dynamically adjusting the damping factor of the pseudo-inverse based on the norm of the current error. This strategy helps to mitigate large joint velocity gains when the target is far from the current position, ensuring smoother control. However, this method proves less effective near singularities, as the error norm does not serve as an adequate damping factor in those situations. Consequently, while ED can be beneficial for general control, it does not fully address the issues posed by singular configurations in robotic manipulators.

To improve the Error Damping (ED) method, the IED algorithm [22] introduces a diagonal matrix $\Omega =\mathrm{diag}(\omega_{1},\cdots ,\omega_{n})$, which adjusts the damping effect more effectively, particularly near singularities. This enhancement ensures better control of the manipulator by modulating the damping based on both the error and system configuration.

In addition, the paper [16] proposes a novel algorithm that filters the Jacobian matrix by selectively modifying its singular values. This approach generates an alternative pseudo-inverse that consistently maintains full rank and ensures a bounded condition number. By doing so, the algorithm improves stability and accuracy, even in the presence of singularities, while avoiding the excessive joint velocity gains associated with conventional pseudo-inverse methods.

The review of methods can be seen in Table 1.

Table 2 summarizes the key advantages and disadvantages of various Jacobian-based inverse kinematics methods for controlling redundant manipulators. Each method offers distinct trade-offs in terms of stability, precision, and handling of singular configurations.

Focusing on the control problem, one of the possible approaches is the Jacobian Weighting (JW) algorithm [24], where a weight matrix W is applied in joint space. This enables prioritization of specific joints for task completion, allowing the controller to assign higher importance to joints nearing their operational limits. When a joint approaches or exceeds its limit, Joint Clamping (JC) [25] can be used to restrict or block the joint’s movement to prevent constraint violations.

Additionally, tasks and their associated Jacobian matrices can be combined using the Augmented Jacobian (JA) method. This concept of task-space augmentation was independently introduced by Sciavicco and Siciliano [26,27,28] and by Egeland [29]. The JA method allows multiple tasks to be incorporated into a single Jacobian representation, enhancing control flexibility. Furthermore, by incorporating task-specific weighting factors into the JA framework, we obtain the Weighted Augmented Jacobian (WJA) method [30].

Another approach, the Gradient Projection (GP) method [31], utilizes the robot’s redundancy to solve secondary tasks by projecting them into the null space of the primary task. This is achieved through the kernel projection operator $N=I-J^{+}J$. By applying this operator, the inverse kinematics Equation (3) is modified to:

(7) $$\dot{q}=J^{*}\dot{x}+Nq_{0},$$

where $q_{0}$ represents a secondary task. This formulation allows the secondary task to be performed without interfering with the primary task, leveraging the robot’s redundancy for more efficient control.

When dealing with multiple tasks, the Task Priority (TP) strategy [32] can be applied. In this approach, the highest-priority task is addressed first, while lower-priority tasks are executed in a manner that does not interfere with the completion of the higher-priority tasks. These lower-priority tasks are projected into the null space of the higher-priority tasks, ensuring that they do not conflict with the primary objective.

One challenge with this strategy, as highlighted in [16,23], is that the pseudo-inverse operator is not continuous, which can lead to abrupt changes in the control behavior. To address this, the authors proposed a method to make the pseudo-inverse operator continuous, resulting in a new strategy called Continuous Task Priority (CTP). This modification ensures smoother task execution by maintaining continuity in the control solution across different task priorities.

The review of methods can be seen in Table 3.

### 2.2. Optimization Approach

The traditional Jacobian-based approach can be computationally expensive in certain scenarios [33]. In this section, an alternative method will be explored, which controls robots without directly computing the inverse kinematics. The key concept behind the optimization approach is the formulation of an objective function that is minimized subject to a set of constraints. This method allows for more flexible control, particularly in complex tasks where inverse kinematics may not be efficient or easily solvable.

However, in the context of an optimization problem, it is typically challenging to make definitive statements about the existence or nature of the solution. Nevertheless, by framing the problem as a convex optimization program, convex optimization theory provides tools to determine both the existence and uniqueness of a solution, given certain conditions [34]. This approach ensures that, under convexity, the solution is not only feasible but also optimal and unique, offering a more structured framework for solving control problems in robotics.

The optimization approach offers a versatile framework for addressing various objectives in robotic control. It allows for minimizing least-square joint velocities [35], maximizing joint range availability [36], and avoiding singularities [37,38]. Recent studies have expanded these applications to include more complex scenarios, such as optimizing energy efficiency [39], improving compliance in human–robot interaction [40], and reducing human fatigue through optimized robot trajectories [41].

This method is also valuable for torque optimization [42] and solving multi-task problems [43], where competing objectives need to be balanced. In recent years, researchers have applied optimization techniques to ensure safe navigation in dynamic environments through real-time trajectory adjustments [44], expanding the method’s applicability to more advanced autonomous systems. Additionally, the approach has proven effective for obstacle avoidance [32,45] and has been enhanced with predictive models to improve planning in uncertain environments [46].

However, before applying this theory, it is essential to introduce a few key definitions to establish the necessary foundation.

**Definition 1.**  *A set $A\subset R^{n}$ is affine, if for every x,y∈A and every λ∈R holds:*

(8) (1−λ)x+λy∈A.

**Definition 2.**  *A set $A\subset R^{n}$ is convex, if for every x,y∈A and every λ∈[0,1] holds:*

(9) (1−λ)x+λy∈A.

From Definitions 1 and 2, it follows that every affine set is also a convex set. The next step is to examine the conditions under which a function is convex and when it is affine.

**Definition 3.**  *Function f:U→V is affine if it holds:*

(10) f((1−λ)x+λy)=(1−λ)f(x)+λf(y),

*for every x,y∈U and λ∈R.*

**Definition 4.**  *Function $f:D\subset R^{n}\to R$ is convex if its domain D is convex set and it holds:*

(11) f((1−λ)x+λy)≤(1−λ)f(x)+λf(y),

*for every x,y∈D and λ∈[0,1].*

In general, optimization problems can be formulated as:

(12) $$\begin{array}{cc} \mathrm{minimize}\; & f_{0}\left(x\right)\\ \mathrm{subject}\;\mathrm{to}:\; & f_{i}\left(x\right)<=0,\;i=1,\;\cdots ,\;m\\ \; & h_{j}\left(x\right)=0,\;j=1,\;\cdots ,\;p. \end{array}$$

In this formulation, $f_{0}\left(x\right)$ represents the objective function, while the functions $f_{i}\left(x\right)$ for i=1,⋯,m and $h_{j}\left(x\right)$ for j=1,⋯,p are the inequality and equality constraints, respectively. These constraints can represent various physical and operational limitations, such as joint rate bounds, joint angle limits, joint acceleration constraints, and other task-specific requirements.

For the optimization program from (12) to qualify as a convex program, the objective function $f_{0}$ and the constraint functions $f_{1},\cdots ,f_{m}$ must be convex functions, while the equality constraint functions $h_{1},\cdots ,h_{p}$ must be affine functions. This ensures that the problem remains tractable and can be efficiently solved using convex optimization techniques.

Convex optimization programs have several useful properties, which can be formally described by the following theorems:

**Theorem 1.**  *In a convex optimization problem as defined in *(12)*, every local minimum is also a global minimum.*

**Proof.**  The proof is provided in [47], page 87. □

**Theorem 2.**  *Consider the convex optimization problem *(12)*. Suppose that $f_{0}$ is strictly convex on the feasible set F. If an optimal solution exists, then it is unique.*

**Proof.**  Suppose there exist two distinct optimal solutions, *x* and *y* (x≠y). This implies that for every z∈F, the following holds:

(13) f(x)=f(y)≤f(z).

Now, let $z=\frac{1}{2}\left(x+y\right)$. Since the set F is convex, it follows that z∈F. Given that $f_{0}\left(x\right)$ is strictly convex, we have:

(14) $$f\left(z\right)=f\left(\frac{1}{2}x+\frac{1}{2}y\right)<\frac{1}{2}f\left(x\right)+\frac{1}{2}f\left(y\right)=f\left(x\right).$$

This leads to a contradiction, which proves the uniqueness of the solution. □

**Theorem 3.**  *In a convex optimization problem *(12)*, if $f_{0}$ is continuously differentiable, then x is an optimal solution if and only if x is feasible and satisfies the following condition:*

(15) $$\nabla f_{0}{\left(x\right)}^{T}\left(y-x\right)\geq 0,$$

*for all y∈F.*

**Proof.**  The proof is detailed in [34], page 139, under Section 4.2.3: “An optimality criterion for differentiable ${f_{0}}^{\prime \prime }$. □

The result of Theorem 3 implies that, for a convex optimization problem of the form

(16) $$\begin{array}{cc} \mathrm{minimize}\; & f_{0}\left(x\right)\\ \mathrm{subject}\;\mathrm{to}:\; & Ax=b, \end{array}$$

where $f_{0}$ is continuously differentiable convex function and $A\in R^{p\times n}$, $b\in R^{p}$. Then, the $x^{*}$ is an optimal solution if and only if, there exists $v^{*}\in R^{p}$, that it holds

(17) $$Ax^{*}=b\;\mathrm{and}\;▽f_{0}\left(x^{*}\right)+A^{T}v^{*}=0.$$

The vector v originates from Lagrange dual theory, which will not be detailed here but is thoroughly explained in [34].

For example, consider an optimization problem of the form where P≻0:

(18) $$\begin{array}{cc} \mathrm{minimize}\; & \frac{1}{2}x^{T}Px+q^{T}x\\ \mathrm{subject}\;\mathrm{to}:\; & Ax=b, \end{array}$$

where $A\in R^{p\times n}$ and $b\in R^{p}$. Let assume that rank r(A)=p<n. Then, it follows from Equation (17) that $x^{*}\in R^{n}$ is the optimal solution if and only if there exists $v^{*}\in R^{p}$ such that the following is true:

(19) $$Ax^{*}=b\;\mathrm{and}\;Px^{*}+q+A^{T}v^{*}=0.$$

This implies that the optimal solution can be obtained by solving a system of n+p linear equations of the form

(20) $$\left[\begin{array}{cc} P & A^{T}\\ A & 0 \end{array}\right]\left[\begin{array}{c} x^{*}\\ v^{*} \end{array}\right]=\left[\begin{array}{c} -q\\ b \end{array}\right],$$

for n+p parameters. The system (20) can result in three different cases:

- If the matrix is invertible, a unique solution for $x^{*}$ is obtained.
- If the matrix is singular but the system is solvable, any solution is considered optimal.
- If the system is not solvable, the optimization problem is either unbounded or infeasible.

The following statements are known to be equivalent:

1. The matrix $\left[\begin{array}{cc} P & A^{T}\\ A & 0 \end{array}\right]$ is non-singular.
2. $Ax=0,x\neq 0⟹x^{T}Px>0$.
3. $P+A^{T}A≻0$.

In summary, the outcome of the optimization problem is determined by the selection of the matrix P. By choosing P to be positive definite, the program ensures a unique optimal solution, provided a feasible solution exists. This choice guarantees that the problem is well-posed, preventing issues such as singularity or non-uniqueness in the solution.

Optimization approaches provide significant advantages by offering a wide range of methods for solving problems, either analytically or through iterative techniques. For unconstrained convex optimization problems, two widely used methods are the gradient descent method and Newton’s method

The gradient method is a versatile tool for iteratively approaching a minimum. Starting from the current approximation $x^{\left(k\right)}$, it selects a direction vector $\Delta x^{\left(k\right)}\in R^{n}$ and updates the next approximation according to

(21) $$x^{\left(k+1\right)}=x^{\left(k\right)}+t^{\left(k\right)}\Delta x^{\left(k\right)},$$

where $t^{\left(k\right)}\geq 0$ represents the step size. The goal is to reduce the value of the objective function *f* at each iteration, ensuring that $f\left(x^{\left(k+1\right)}\right)<f\left(x^{\left(k\right)}\right)$. The choice of $t^{\left(k\right)}$ determines the specific variant of the method, with common strategies including exact line search or backtracking line search techniques [34,48].

Newton’s method takes a more advanced approach to optimization compared to gradient-based methods. If the function *f* is quadratic, the optimal solution can be determined by updating the current point *x* using the Newton step $\Delta x_{nt}$, where

(22) $$\Delta x_{nt}=-\nabla^{2}f{\left(x\right)}^{-1}\nabla f\left(x\right).$$

In regions near the current approximation *x*, the function *f* can be closely approximated by a quadratic function $\hat{f}$, and the minimum of $\hat{f}$ determines the next approximation. As the solution approaches the optimal point $x^{*}$ (where ∇f(x)≈0), $\hat{f}$ becomes a more accurate representation of *f*. Additional details on this method can be found in the literature [34,49].

For optimization problems with linear constraints or inequalities, interior-point methods provide effective solutions, as detailed in [34]. Popular variants include interior-point barrier methods and primal-dual interior-point methods, each tailored to specific problem structures.

Additionally, AI-based methods such as genetic algorithms [50,51] and neural networks, as discussed in [52], offer alternative approaches for solving optimization challenges, particularly in complex domains like robotic manipulator modeling and control.

## 3. Exploiting Leaning on Surface for Accurate End-Effector Motion

In robotic manipulation, executing complex tasks often requires the ability to manage multiple objectives simultaneously. One such scenario is the interaction of a robot with external surfaces, where both stability and dynamic motion are critical. By leaning the robot mechanism onto a stable surface, the system can leverage the external force for enhanced stability, allowing for more efficient execution of secondary tasks, such as dynamic end-effector motion. This two-task approach is not only relevant for practical applications but also challenges the robot’s ability to control both contact forces and motion.

Previous sections reviewed various optimization and control methods, including task-priority strategies, which are well-suited for handling multi-task problems. In this context, the concept of surface leaning can be framed as a specific instance of a two-task control problem, where force control and dynamic motion must be managed concurrently. This paper explores the application of these control strategies to achieve desired outcomes in such scenarios.

**Figure 1:**
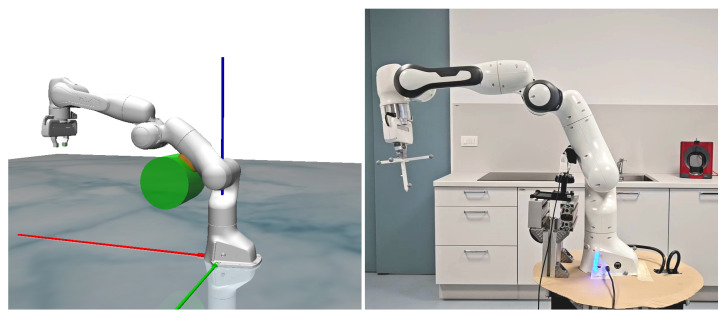
The concept of leaning the robot mechanism on a fixed surface.

The concept involves the robot leaning against a surface while maintaining a desired force, $F_{d}$, and simultaneously performing dynamic motion with its end effector. Either task can be prioritized depending on the specific application. This concept is illustrated in Figure 1.

To illustrate the first task, we simplified the scenario without loss of generality. In this case, the objective is for the robot mechanism to exert a force on an object using the segment between the second and third joints. Consequently, this task is exclusively related to the control of the first and second joints, with the corresponding Jacobian matrix taking the following form

(23) $$J_{f}=\left[\begin{array}{ccccccc} 1 & 0 & 0 & 0 & 0 & 0 & 0\\ 0 & 1 & 0 & 0 & 0 & 0 & 0 \end{array}\right].$$

It is crucial to emphasize that the Jacobian matrix in this scenario is non-invertible. To address this issue, we apply the Moore–Penrose pseudo-inverse, which yields

(24) $$J_{f}^{+}={\left[\begin{array}{ccccccc} 1 & 0 & 0 & 0 & 0 & 0 & 0\\ 0 & 1 & 0 & 0 & 0 & 0 & 0 \end{array}\right]}^{T}.$$

Please note that the resulting pseudo-inverse, represented as $J_{f}^{+}$, is essentially the transpose of the original Jacobian matrix $J_{f}$.

There are two possible approaches to solve this first task. Assuming the target force magnitude is denoted by $F_{d}$, the necessary adjustment to achieve the desired force can be computed as $e_{f}^{\prime }=F_{d}-F$. Based on the experimental setup illustrated in Figure 1 and considering the robot’s kinematics, it becomes evident that the first joint cannot generate the required vertical force. Given this limitation, the approach can be simplified by setting the first component of the vector $e_{f}$ to zero without loss of generality. Considering the significant influence of the second joint on the leaning force, the vector $e_{f}$ can be effectively represented as $e_{f}={\left[0,\;e_{f}^{\prime }\right]}^{T}$.

The second option for addressing the leaning task is to use a weighted pseudo-inverse. This method enables control over which joints contribute to the leaning task and to what extent, by selecting appropriate weights. As a result, this approach can be applied to a variety of leaning tasks, where multiple joints are involved in generating the desired force, allowing for more flexible and efficient task execution.

To enable the robot to perform an additional task, a controller can be defined as follows

(25) $${\dot{q}}_{d}=J_{f}^{+}K_{f}e_{f}+N_{f}J_{x}^{*}K_{x}e_{x},$$

where $N_{f}$ represents the null-space projection of $J_{f}$. Additionally, $K_{f}$ and $K_{x}$ denote the proportional gains for the force-feedback and Cartesian controllers, respectively. Furthermore, $J_{x}^{*}$ is defined as an approximation of the inverse of the Jacobian matrix $J_{x}$ for the secondary task.

The first task has already been addressed. The focus now shifts to solving the second task. As explained in earlier sections, two primary approaches are available for this problem: the first approach utilizes inverse kinematics methods, while the second employs quadratic programming techniques. Each approach provides distinct advantages based on the structure and requirements of the task.

### 3.1. Gradient Projection and Jacobian Pseudo Inverse

This method enables a robotic mechanism to solve multi-task problems in a hierarchical manner. The primary task is prioritized and solved first, while the secondary task is addressed within the null space of the primary task. This hierarchical approach ensures that the secondary task does not interfere with the performance of the primary task, allowing for efficient multi-task execution.

In our use case scenario, the primary task involves leaning on a surface and maintaining a force of $F_{d}=10\;N$. The Jacobian matrix for this task, as defined in Equation (23), is used to compute the null space via the projection operator $N=I-J^{+}J$. Given that the Jacobian matrix $J_{f}$ has dimensions 2×7, the resulting null space matrix N will have dimensions 7×7, corresponding to the robot’s 7 degrees of freedom (DOF). This null-space projection allows for executing secondary tasks without affecting the primary force control task.

In this scenario, the Jacobian Pseudo-inverse method is applied to solve the second task. Let $x_{d}$ represent the desired pose of the robotic mechanism and x denote its current pose. The vector $e=x-x_{d}$ represents the pose error, or the required adjustment to the pose for task completion.

By utilizing the inverse kinematics Equation (3) and approximating the inverse of the Jacobian matrix J for the second task with its pseudo-inverse, the problem is addressed. This approach ensures that the pose error is minimized while maintaining the constraints of the primary task.

It is crucial to emphasize that the success of solving the secondary task is contingent upon the mechanism’s ability to first accomplish the primary task. As a result, the choice of the primary task is of paramount importance in this method, as it directly impacts the feasibility and effectiveness of executing the secondary task within the null space.

### 3.2. Gradient Projection and Jacobian Weighting

The rationale behind employing the weighting method is to improve upon the standard pseudo-inverse approach. The standard pseudo-inverse typically selects a solution that minimizes the second norm, resulting in smoother movements by assigning equal importance to all joints. By applying weights, the Jacobian Weighting method allows for prioritization of specific joints, enhancing the control flexibility in executing the secondary task.

However, a drawback of the standard pseudo-inverse approach is that it considers all joints equally, regardless of their relevance to the primary task. To address this, the Jacobian Weighting method is introduced, which employs the Jacobian matrix J of the secondary task, weighted by the null space of the primary task. This ensures that the solution for the secondary task exclusively utilizes joints that do not interfere with the primary task. The Jacobian Weighting method is structured as follows

(26) $$J_{x}^{+}=N_{f}^{-1}J_{x}^{T}{\left(J_{x}N_{f}^{-1}J_{x}^{T}+\mu I\right)}^{-1}.$$

The Jacobian Weighting method generates solutions that do not interfere with the primary task, offering superior accuracy compared to the standard pseudo-inverse method. By prioritizing joints that have minimal impact on the primary task, this method enhances the precision of the robot’s end-effector placement. As a result, it optimizes performance for secondary tasks while ensuring that the objectives of the primary task are not compromised.

### 3.3. Augmented Jacobian

If task prioritization is not desired, the Augmented Jacobian method can be employed, allowing both tasks to be solved simultaneously. In some versions of this method, weights can be assigned to specify the relative importance of each task; however, both tasks will still be addressed concurrently. The first step is to combine the error vectors e for both tasks, which can be performed by concatenating them into a matrix

(27) $$e_{a}=\left[\begin{array}{c} e_{f}\\ e \end{array}\right].$$

Next, the same procedure needs to be applied to the Jacobian matrices

(28) $$J_{a}=\left[\begin{array}{c} J_{f}\\ J \end{array}\right].$$

Since the kinematic equations can be also applied for matrices $e_{a}$ and $J_{a}$, by using inverse kinematic Equation (3). This formulation ensures that both tasks are solved simultaneously, incorporating the kinematic constraints from each task into a unified solution.

### 3.4. Quadratic Programming

In solving robotic control problems, selecting an appropriate objective function is crucial for optimizing system performance. The choice of the objective directly impacts the smoothness and efficiency of the robot’s motion.

One potential choice for the objective function $f_{0}$ is the norm of the velocity vector, $∥\dot{q}∥$. Minimizing this norm results in reduced joint velocities, leading to smoother movement in the robotic system. Since the norm is a positive convex function, as derived from the triangle inequality, multiplying it by any positive function preserves its convexity. Therefore, an alternative objective function can be formulated as the squared norm, $\frac{1}{2}{∥\dot{q}∥}_{2}^{2}$. This form is beneficial as it allows the norm to be expressed in terms of vectors and matrices, specifically

(29) $${∥\dot{q}∥}_{2}^{2}=\sum_{i=1}^{n}{\left|{\dot{q}}_{i}\right|}^{2}=\dot{q}\;I\;\dot{q},$$

where I represents the identity matrix. Equation (29) represents a quadratic function, which allows the problem to be formulated as a quadratic programming problem, as described in (18). By using the identity matrix I, which is positive definite by definition, for the matrix P, it ensures that the optimization problem will have a unique optimal solution, provided that a solution exists. This choice of P guarantees that the program remains convex and efficiently solvable.

Focusing on the constraints, there are several ways to define them. In the first scenario, the constraints are set similarly to the Augmented Jacobian method, where the kinematic equations for both tasks are used as constraints. Specifically, the first constraint takes the form $J_{f}\dot{q}=e_{f}$, and the second constraint is $J\dot{q}=e$. These constraints can also be combined into a single form as

(30) $$J_{a}\dot{q}=e_{a},\;\mathrm{where}\;J_{a}=\left[\begin{array}{c} J_{f}\\ J \end{array}\right],\;\mathrm{and}\;e_{a}=\left[\begin{array}{c} e_{f}\\ e \end{array}\right].$$

That gives us a quadratic program of the form:

(31) $$\begin{array}{cc} \mathrm{minimize}\; & \frac{1}{2}\dot{q}\;I\;\dot{q}\\ \mathrm{subject}\;\mathrm{to}:\; & J_{f}\dot{q}=e_{f},\\ \; & J\dot{q}=e. \end{array}$$

By using Theorem 3 this program can be rewritten into a system of linear equations:

(32) $$\left[\begin{array}{cc} I & J_{a}^{T}\\ J_{a} & 0 \end{array}\right]\left[\begin{array}{c} {\dot{q}}^{*}\\ v^{*} \end{array}\right]=\left[\begin{array}{c} 0\\ e_{a} \end{array}\right].$$

If the system of linear Equation (32) is solved analytically, the obtained solution would be identical to that of the Augmented Jacobian method. This equivalence is evident from

(33) $$\begin{array}{cc} \; & I\dot{q}+J_{a}^{T}v=0\Rightarrow \dot{q}=-J_{a}^{T}v,\\ \; & J_{a}\dot{q}=e_{a}\Rightarrow -J_{a}J_{a}^{T}v=e_{a}\Rightarrow v=-{\left(J_{a}J_{a}^{T}\right)}^{-1}e_{a},\\ \; & \dot{q}=J_{a}^{T}{\left(J_{a}J_{a}^{T}\right)}^{-1}e_{a}=J^{+}e_{a}. \end{array}$$

In the context of quadratic optimization problems, various methods can be adapted to meet specific requirements. One such approach involves formulating a quadratic program, similar to the Jacobian Weighted method, where weights are assigned to prioritize certain joint movements over others. Before implementing this approach, it is crucial to define the norm that will be minimized, as it serves as the foundation for optimizing the robot’s movements while maintaining task performance.

**Theorem 4.**  *Function ${∥\cdot ∥}_{W}:R^{n}\to R$, for W≻0, defined as*

(34) $${∥x∥}_{W}=\sqrt{x^{T}Wx},$$

*is norm.*

**Proof.**  To demonstrate that the function from Equation (34) is a valid norm, it must satisfy the following three conditions:

1. Positive definiteness: for all $x\in R^{n}$ the ${∥x∥}_{W}\geq 0$, and if ${∥x∥}_{W}=0$, then x=0.
2. Absolute homogeneity: ${∥\alpha x∥}_{W}={|\alpha |∥x∥}_{W}$, for all $x\in R^{n}$ and α∈R.
3. Subadditivity/Triangle inequality: ${∥x+y∥}_{W}\leq {∥x∥}_{W}+{∥y∥}_{W}$ for all $x,\;y\in R^{n}$.

It is obviously that ${∥x∥}_{W}\geq 0$, and since the matrix W is positive definite, it also holds ${∥x∥}_{W}=0$ if and only if x=0.

The second condition follows from:

(35) $${∥\alpha x∥}_{W}=\sqrt{{\left(\alpha x\right)}^{T}W\left(\alpha x\right)}=\left|\alpha \right|\sqrt{x^{T}Wx}={|\alpha |∥x∥}_{W}.$$

For the third condition, the matrix W must be positive definite. This requirement ensures the existence of an orthogonal matrix Q and a diagonal matrix D with positive diagonal elements. Consequently, W can be expressed as $W=QDQ^{T}$.

Additionally, $W^{\frac{1}{2}}$ can be defined as $W^{\frac{1}{2}}=QD^{\frac{1}{2}}Q^{T}$, where $D{ii}^{\frac{1}{2}}=\sqrt{dii}$. From this, it follows that

(36) $$\begin{array}{cc} {∥x+y∥}_{W}^{2} & ={\left(x+y\right)}^{T}W\left(x+y\right)\\ \; & =x^{T}Wx+y^{T}Wy+2x^{T}Wy\\ \; & ={∥x∥}_{W}^{2}+{∥y∥}_{W}^{2}+2x^{T}W^{\frac{1}{2}}W^{\frac{1}{2}}y\\ \; & \leq {∥x∥}_{W}^{2}+{∥y∥}_{W}^{2}+2∥W^{\frac{1}{2}}{x∥}_{2}{∥W^{\frac{1}{2}}y∥}_{2}\\ \; & ={∥x∥}_{W}^{2}+{∥y∥}_{W}^{2}+{2∥x∥}_{W}{∥y∥}_{W}\\ \; & ={(∥x∥}_{W}+{∥y∥}_{W}{)}^{2}. \end{array}$$

In this derivation, the Cauchy–Schwarz inequality was applied. By taking the square root of the resulting expression, the triangle inequality is obtained. Thus, the third condition is satisfied, completing the proof that the function defined in Equation (34) is indeed a norm. □

Assume a system with two states connected through the kinematic Equation (1). Now, suppose the goal is to minimize the weighted norm of $\dot{x}$ using an objective function. This can be expressed as

(37) $${∥\dot{x}∥}_{W}^{2}={\dot{x}}^{T}W\dot{x}={\left(J\dot{q}\right)}^{T}W\left(J\dot{q}\right)={\dot{q}}^{T}J^{T}WJ\dot{q}={∥\dot{q}∥}_{J^{T}WJ}^{2}.$$

From this, it follows that a quadratic program can be formulated in the following form

(38) $$\begin{array}{cc} \min_{\dot{q}}\; & \frac{1}{2}{\dot{q}}^{T}\left(J^{T}WJ\right)\dot{q}\\ \mathrm{subject}\;\mathrm{to}:\; & A\dot{q}=b\\ \; & E\dot{q}\leq d, \end{array}$$

where A, b, E, and d represent the equality and inequality constraints, respectively.

## 4. Evaluation, Results, and Comparison of Multiple Methods

To compare the performance of different control strategies, a predefined trajectory in the form of an infinity symbol (*∞*) with a specified radius *r* was established as a reference path. The deviation from this trajectory was measured to quantify the error during movement, providing a clear and consistent metric for evaluating the precision of each control approach. To extend the motion into three-dimensional space, the trajectory was parameterized to include a continuously varying third coordinate. The resulting parametric representation of this curve, defined for any t∈R, is expressed by

(39) $$x_{d}\left(t\right)=\left[\begin{array}{c} r\cdot \sin(t)\cdot \cos(t)+0.65\\ r\cdot \cos(t)\\ r\cdot \sin\left(t\right)\cdot \cos^{2}\left(t\right)+0.4 \end{array}\right].$$

**Figure 2:**
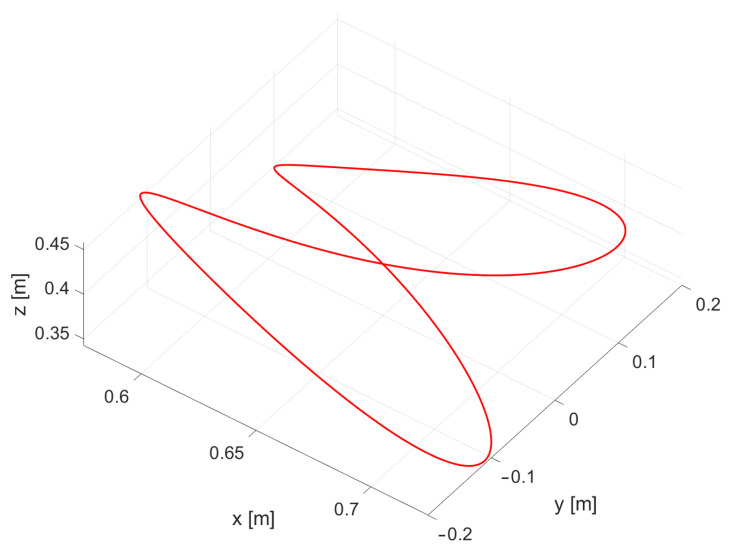
Trajectory of the parametric function (39) over the interval t∈[0,2π], illustrating the complete path of the infinity-shaped curve used for evaluating control precision.

For *t* in the interval [0,2π], the function generates the curve depicted in Figure 2.

To evaluate and compare the efficacy of the proposed methods, where the robot maintains its structure by making contact with a surface, the performance is contrasted against a traditional approach in which no contact is made. These tests are carefully designed to emphasize the key differences in performance between the two methodologies, allowing for a comprehensive assessment of their respective advantages and limitations.

In all scenarios, the robot operates with 7 degrees of freedom (DOF) and is programmed to exhibit increased flexibility in its first two joints. This flexibility is achieved by implementing impedance control and setting the internal gains of the first two joints to low values, effectively simulating mechanisms with high passive compliance. This aspect is crucial for assessing the robot’s precision, especially in simpler tasks where its precision capabilities or limitations can be more distinctly observed. This feature plays a pivotal role in the proposed method, as the robot is required not only to execute precise movements but also to exert a constant, controlled force, making it a key factor in evaluating the method’s overall effectiveness.

The dual functionality—movement and force application—tests the robot’s ability to maintain stability and accuracy under varying operational conditions. The additional requirement of force application allows an evaluation of how it influences the robot’s overall performance, especially in terms of precision and adaptability. By comparing these two approaches, the goal is to assess the robot’s functional capabilities, adaptability, and reliability in different operational contexts. This comparison ultimately reveals the advantages and limitations of each method.

Furthermore, the experiment critically examines the impact of maintaining a constant force $F_{d}=10\;N$ using the proposed methods on the overall precision of the end effector. This is compared to the basic method, where the robot operates without the additional force requirement. The comparative analysis quantifies accuracy using the error estimator $e_{r}$, which is defined as:

(40) $$e_{r}=∥\left(x-x_{d}\right){∥}_{2}+∥\left(y-y_{d}\right){∥}_{2}+{∥\left(z-z_{d}\right)∥}_{2},$$

where $x_{d},y_{d},z_{d}$ represent the desired positions, and x,y,z are the actual positions of the end effector. This error metric provides a comprehensive measure of the deviation in all spatial dimensions, enabling a precise comparison of the proposed methods in terms of end effector accuracy. The standard deviation will be calculated by:

(41) $$e_{sd}=sd\left(x-x_{d}\right)+sd\left(y-y_{d}\right)+sd\left(z-z_{d}\right),$$

where sd represents an operator $sd\left(X\right)=\sqrt{E\left[X^{2}\right]-E{\left[X\right]}^{2}}$.

All methods were implemented on the Franka Emika Panda robot. For each method, the control loop execution time and the average error of the end effector over one complete period were measured. The results are summarized in Table 4.

**Figure 3:**
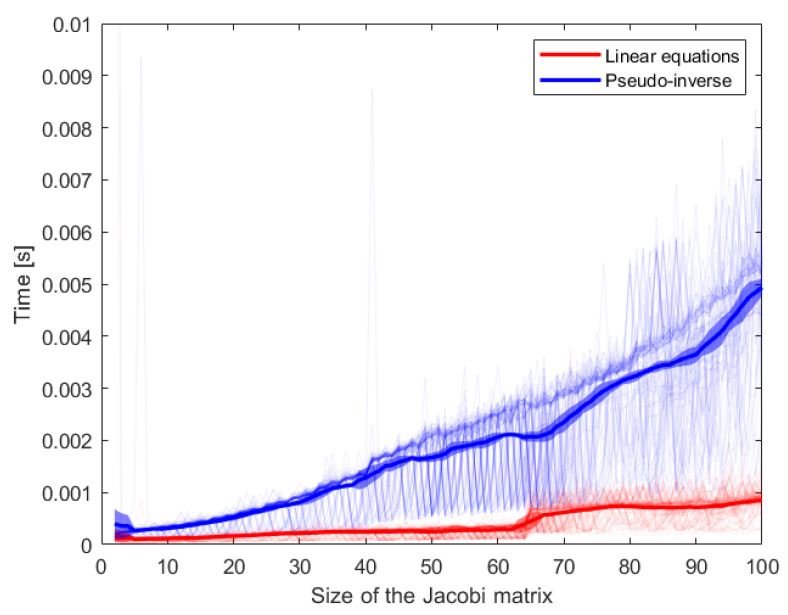
Comparison of the time required to compute the pseudo-inverse of a matrix and the time needed to solve a system of linear equations as a function of the system size. Each computation was repeated 100 times, and the bold line represents the mean time for each method across different sizes. The standard deviation at each size is also included.

In Table 4, the data show that methods utilizing analytical solutions exhibit similar average control loop times, whereas quadratic programming is considerably slower. This increased average time for the control loop is attributed to the iterative nature of quadratic programming, which tends to be more time-consuming than analytical methods. However, as the size of the robot and, consequently, the Jacobian matrix increase, quadratic programming can become more efficient than the analytical solution, especially when the quadratic program is converted into a system of linear equations. Figure 3 presents a comparison between the time required to compute the pseudo-inverse and the time needed to solve the system of linear equations as a function of matrix size, measured on an Intel Core i7 CPU.

Focusing on the errors of these methods, it is observed that quadratic programming exhibits the highest error among the evaluated approaches. However, this error can be mitigated through careful fine-tuning and appropriate parameter adjustments. By reducing the distance from the optimal solution, the error can be minimized, though this comes at the expense of increased computational time. The iterative nature of quadratic programming requires more iterations to converge to a solution that is closer to the optimum, thereby enhancing accuracy. Consequently, while the accuracy of quadratic programming can be improved, it involves a trade-off between error reduction and computational efficiency.

The gradient projection and Jacobian pseudo-inverse methods exhibit the second-highest error. This is due to the way these methods handle the dual-task requirement. Initially, they determine the configuration needed for the robot to lean on a surface, specifically by calculating the angles of the first two joints. When performing the secondary task, the methods compute the desired configuration using all seven joints. However, this solution is subsequently multiplied by the null space of the primary task, which introduces additional discrepancies and results in a larger error. The interaction with the null space effectively compromises the accuracy of the secondary task, leading to a higher overall error.

**Figure 4:**
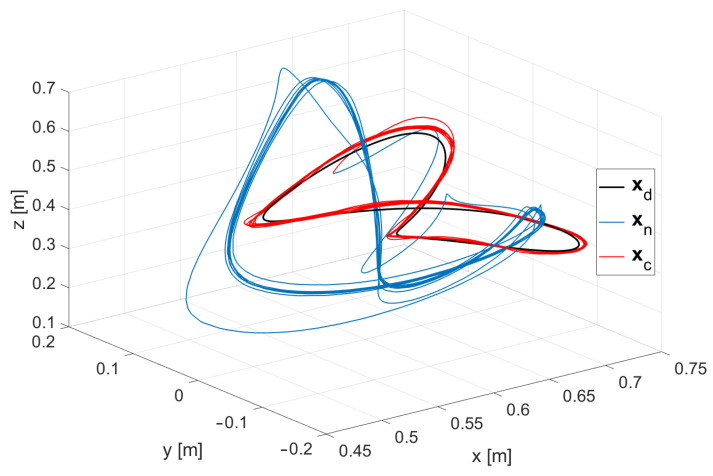
Trajectories of the end effector in cases where the robot was leaning $x_{c}$ (red) and not leaning $x_{n}$ (blue). The black trajectory $x_{d}$ represents the desired motion path as defined by the target equation. The comparison highlights the increased accuracy achieved through the leaning mechanism.

**Figure 5:**
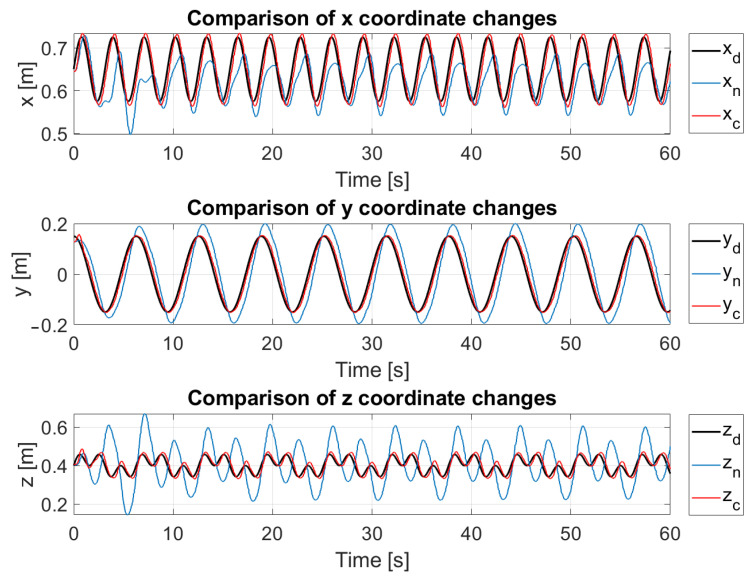
Comparison by coordinates. Black curves represent goal motion, red ones represent the mechanism when it was leaned on the fixed surface, and blue ones represent the mechanism when it was not leaned.

**Figure 6:**
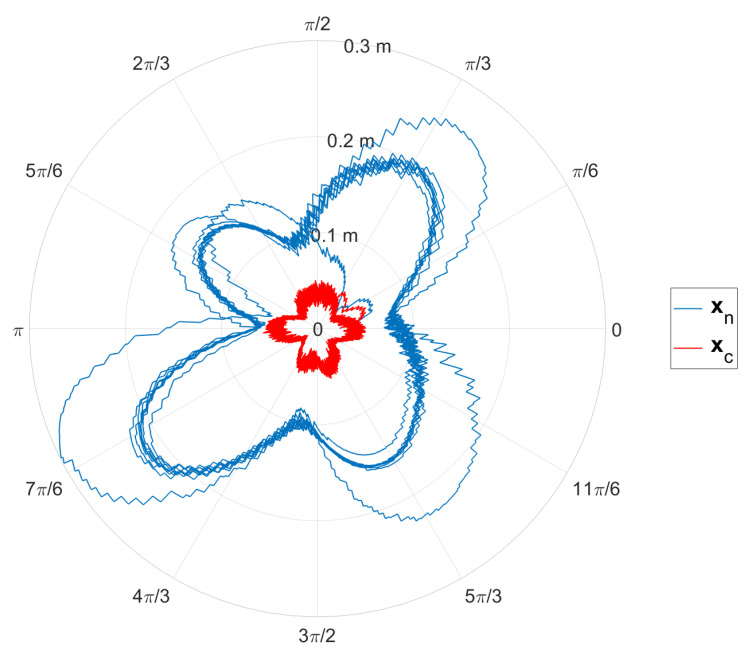
End effector error in polar coordinates. The blue line, $x_{n}$, represents the standard approach, while the red line, $x_{c}$, represents our proposed method.

Figure 4 presents the results of implementing the method that utilizes Jacobian Weighting, compared against a traditional approach that does not incorporate the leaning concept. In this figure, the blue curves represent the traditional approach, the red curves depict the proposed method, and the black curves correspond to the trajectory defined by Equation (39). The results indicate that incorporating the leaning concept significantly improved the accuracy of the robot’s end effector. Additionally, Figure 5 provides a coordinate-wise comparison, showing that the largest error occurred along the *z*-axis. This error was notably reduced by employing the leaning technique, which leveraged the fixed surface to enhance overall precision. Figure 6 illustrates the comparison between the errors of the standard approach and our proposed method. As shown in the figure, our method significantly reduces the error.

## 5. Discussion and Conclusions

This research presented a comprehensive range of methods for controlling robotic mechanisms, culminating in the development of the proposed contributions. Various strategies for addressing inverse kinematics problems were explored, emphasizing that when dealing with multiple tasks, one can either employ the null space approach or integrate all tasks into a unified optimization framework. This provided a foundation for understanding how to manage task priorities and maintain precise end-effector positioning, which is crucial in multi-objective robotic control scenarios.

The primary contribution of the proposed approach focused on leveraging optimization techniques to enhance the robot’s accuracy, particularly when additional support is required by leaning on a surface. Traditional methods often rely on the pseudo-inverse for solving inverse kinematics; however, this can be computationally demanding, especially for robots with a large number of joints. By using optimization methods, specifically quadratic programming, the proposed approach offers an alternative that avoids the complexities associated with pseudo-inverse calculations. This choice was motivated by the need for a more efficient and scalable solution that maintains precision without the computational overhead typically encountered with high-DOF robotic systems.

While AI-based approaches, such as neural networks and fuzzy systems, have been increasingly applied to solving quadratic optimization problems, as reviewed in [52], they were not adopted in the proposed method. This decision was based on the objective of developing a solution that is generalizable and adaptable across different robotic systems without the necessity for extensive training or system-specific tuning. AI-based methods often require large amounts of training data and computational resources to fine-tune models for specific systems or tasks, which can be a significant limitation when aiming for flexibility across multiple environments. Instead, our approach focused on employing well-established quadratic and linear programming techniques, which provide stable, accurate control solutions without the added complexity of machine learning models. These methods offer the benefit of being immediately applicable to various robotic tasks without the need for retraining, making them more suitable for dynamic, real-world scenarios.

The results demonstrated that the proposed method significantly improved precision when the robot employed surface contact to enhance stability and accuracy. By incorporating this leaning mechanism, the approach effectively reduced errors in end-effector positioning, validating the efficacy of the optimization framework in practical applications. Comparative analysis with traditional methods highlighted the advantages of using optimization techniques, particularly in scenarios requiring precise force application and positional accuracy. This demonstrates the robustness and adaptability of the proposed approach in addressing the challenges of multi-task robotic control.

For future work, it is recommended to investigate how the leaning concept can be extended beyond the robot’s internal structure to interact with external objects and environments. While this study primarily focused on improving accuracy through leaning, physical contact with the surroundings could potentially enhance other aspects of robotic performance. For instance, leveraging external supports could increase the robot’s payload capacity, enabling it to handle heavier objects more efficiently. This approach would be particularly advantageous in scenarios where a robot needs to manipulate a heavy object, such as lifting one side of a large stick. By strategically using external supports, the robot could gradually lift heavier loads that exceed its standard capacity. Extending the leaning concept in this manner could unlock new possibilities for improving the functionality and adaptability of robotic systems in various operational contexts.

## Figures and Tables

**Table 1:** Summary of methods for handling singularities and improving control in redundant manipulators. The table is adapted from [16,23].

Name	Abb.	Equation ($\dot{q}$=)	Ref.
Jacobian Pseudo-inverse	JP	$J^{+}\dot{x}=\sum_{i=1}^{n}\frac{1}{\sigma_{i}}v_{i}\left(u_{i}^{T}\cdot \dot{x}\right)$	[16]
Jacobian Transpose	JT	$J^{T}\dot{x}=\sum_{i=1}^{n}\sigma_{i}v_{i}\left(u_{i}^{T}\cdot \dot{x}\right)$	[17]
Selective Damping	SD	$J^{+SD}\dot{x}=\sum_{i=1}^{n}s\left(\sigma_{i},i,J,\gamma_{max}\right)v_{i}\left(u_{i}^{T}\cdot \dot{x}\right)$	[18]
Damped Jacobian	JD	$\begin{array}{cc} J^{+D}\dot{x} & =J^{T}{\left({\mathrm{JJ}}^{T}+\lambda^{2}I\right)}^{-1}\dot{x}=\sum_{i=1}^{n}\frac{\sigma_{i}}{\sigma_{i}^{2}+\lambda^{2}}v_{i}\left(u_{i}^{T}\cdot \dot{x}\right) \end{array}$	[19]
Filtered Jacobian	JF	$\begin{array}{cc} J^{+F}\dot{x} & =J^{T}{\left({\mathrm{JJ}}^{T}+\lambda^{2}u_{n}u_{n}^{T}\right)}^{-1}\dot{x}=\\ & =\sum_{i=1}^{n}\frac{1}{\sigma_{i}}v_{i}\left(u_{i}^{T}\cdot \dot{x}\right)+\frac{\sigma_{n}}{\sigma_{n}^{2}+\lambda^{2}}v_{n}\left(u_{n}^{T}\cdot \dot{x}\right) \end{array}$	[20]
Error Damping	ED	$\begin{array}{cc} J^{+ED}\dot{x} & =J^{T}{\left({\mathrm{JJ}}^{T}+{\mathrm{EI}}_{n}\right)}^{-1}\dot{x}=\sum_{i=1}^{n}\frac{\sigma_{i}}{\sigma_{i}^{2}+E}v_{i}\left(u_{i}^{T}\cdot \dot{x}\right) \end{array}$	[21]
Improved Error Damping	IED	$\begin{array}{cc} J^{+IED}\dot{x} & =J^{T}{\left({\mathrm{JJ}}^{T}+{\mathrm{EI}}_{n}+\Omega \right)}^{-1}\dot{x}=\sum_{i=1}^{n}\frac{\sigma_{i}}{\sigma_{i}^{2}+E+\omega_{i}}v_{i}\left(u_{i}^{T}\cdot \dot{x}\right) \end{array}$	[22]
Singular Value Filtering	SVF	${\tilde{J}}^{+}\dot{x}=\sum_{i=1}^{n}\frac{\sigma^{2}+v\sigma +2}{\sigma^{3}+v\sigma^{2}+2\sigma +2\sigma_{0}}v_{i}\left(u_{i}^{T}\cdot \dot{x}\right)$	[16]

**Table 2:** Summary of advantages and disadvantages of different Jacobian-based methods for inverse kinematics control.

Method	Advantages	Disadvantages
Jacobian Pseudo-inverse (JP)	Solves the minimization problem effectively for redundant manipulators.	Produces large joint velocities near singularities, leading to numerical instability.
Jacobian Transpose (JT)	Avoids the large velocity gains of JP near singularities.	Suffers from conditioning issues similar to JP, especially near singular configurations.
Selective Damping (SD)	Reduces joint velocities specifically in problematic directions without affecting all directions.	Does not fully address the rank loss near singularities; precision is reduced in damped directions.
Damped Jacobian (JD)	Increases stability near singularities by adding a small damping term.	Reduces overall accuracy due to the uniform increase in all singular values.
Filtered Jacobian (JF)	Adaptive damping near singularities, improving control in those situations.	Can still result in significant precision loss near small singular values.
Error Damping (ED)	Reduces large joint velocities when the target is far away, ensuring smoother control.	Ineffective near singularities, as the error norm is not sufficient to handle instability in those cases.
Improved Error Damping (IED)	Better damping adjustment near singularities, improving control stability.	More complex to implement, and still struggles in extreme singular configurations.
Singular Value Filtering (SVF)	Maintains full rank and ensures bounded condition numbers, improving stability near singularities.	Precision may be reduced when operating close to very small singular values.

**Table 3:** Overview of control methods for controlling redundant manipulators. The table is adapted from [16,23].

Name	Abbreviation	Equation	References
Jacobian Weighting	JW	$\dot{q}=W^{-1}J^{T}{\left({\mathrm{JW}}^{-1}J^{T}\right)}^{-1}\dot{x}$	[24]
Gradient Projection	GP	$\dot{q}=J^{*}\dot{x}+Nq_{0}$	[31]
Joint Clamping	JC	$\dot{q}=H{\left(\mathrm{JH}\right)}^{+}\dot{x}$	[25]
Augmented Jacobian	JA	$\dot{q}=J_{v}^{+}\dot{x}$	[26]
Weighted Augmented Jacobian	WJA	$\begin{array}{cc} \dot{q}= & {\left(J_{1}^{T}W_{1}J_{1}+J_{2}^{T}W_{2}J_{2}+\ldots +W_{n}\right)}^{-1}\\ & \left(J_{1}^{T}W_{1}{\dot{x}}_{1}+J_{2}^{T}W_{2}{\dot{x}}_{2}+\ldots \right) \end{array}$	[30]
Task Priority	TP	$\begin{array}{cc} \dot{q} & =-H(\mu \theta )\\ & +{\left[J\left(I_{m}-H^{+}H\right)\right]}^{+}\left(\dot{x}+\mathrm{JH}\left(\mu \Theta \right)\right) \end{array}$	[32]
Continuous Task Priority	CTP	$\dot{q}=H\left(-\mu \theta \right)+J^{{\left(I_{m}-H\right)}^{⨁}}(\dot{x}-\mathrm{JH}\left(-\mu \theta \right)$	[16]

**Table 4:** Implementation results using various methods.

Name	Average Time of the Control Loop	Standard Deviation of the Control Loop Time	Error $e_{r}$ of the End Effector	Standard Deviation of the End-Effector Error
Gradient Projection and Jacobian Pseudo-inverse	0.0022	0.0025	0.4162	0.0076
Gradient Projection and Jacobian Weighting	0.0020	0.0024	0.2754	0.0053
Weighted Augmented Jacobian	0.0023	0.0026	0.3336	0.0063
Quadratic programming	0.0078	0.0050	2.096	0.0741

## Data Availability

The original contributions presented in the study are included in the article, further inquiries can be directed to the corresponding author.

## Abbreviations

The following abbreviations are used in this manuscript:

DOF	Degrees of freedom
SVD	Singular Value Decomposition
JP	Jacobian Pseudo-inverse
JT	Jacobian Transpose
SD	Selective Damping
JD	Jacobian Damping
JF	Filtered Jacobian
ED	Error Damping
IED	Improved Error Damping
SVF	Singular Value Filtering
JW	Jacobian Weighting
JC	Joint Clamping
JA	Augmented Jacobian
WJA	Weighted Augmented Jacobian
GP	Gradient Projection
TP	Task Priority
CTP	Continuous Task Priority
AI	Artificial Intelligence
